# Case report: Successful treatment of hyperkalemia during general anesthesia in a domestic cat

**DOI:** 10.3389/fvets.2024.1398128

**Published:** 2024-08-08

**Authors:** Tiffany Irizarry, Sarah Gradilla

**Affiliations:** ^1^Emergency Critical Care Department, Ocean State Veterinary Specialists, East Greenwich, RI, United States; ^2^Emergency Critical Care Department, DoveLewis Veterinary Emergency & Specialty Hospital, Portland, OR, United States

**Keywords:** creatine kinase, cardiac and pulmonary resuscitation, hyperkalemia, propofol-related infusion syndrome, case report

## Abstract

**Objective:**

This study aimed to describe the successful identification and treatment of severe hyperkalemia, cardiac arrhythmia, rhabdomyolysis, and acute kidney injury (AKI) in a domestic cat that underwent general anesthesia for abdominal exploratory surgery. The definitive underlying cause remains unknown; however, a reaction to propofol is suspected.

**Case summary:**

A 6-month-old intact male domestic short-hair cat underwent general anesthesia and developed severe intraoperative rhabdomyolysis, hyperkalemia, ventricular fibrillation, and AKI during surgery despite a documented mild hypokalemia and normal creatinine before inducing anesthesia. Propofol was administered as part of the anesthetic protocol. The patient was resuscitated successfully and responded well to advanced medical intervention. The hyperkalemia and AKI were resolved within less than 24 h from surgery and rhabdomyolysis was resolved at the time of recheck 5 days later.

**New or unique information provided:**

While previously suspected in dogs, to the authors’ knowledge, propofol-related infusion syndrome (PRIS) has not been reported in domestic cats. Veterinary professionals should be aware that drug-induced intraoperative rhabdomyolysis and hyperkalemia can develop unexpectedly and should remain a differential for acute cardiac arrhythmias or cardiac arrest and AKI.

## Introduction

Propofol is frequently used as an intravenous anesthetic for both sedated and general anesthesia protocols in human and veterinary medicine. Despite being considered a safe and reliable anesthetic agent, a rare, life-threatening complication called propofol-related infusion syndrome (PRIS) has been documented in human medicine (1–6). While no definition has been universally accepted and the pathophysiology is not clearly understood, PRIS presents a constellation of clinical features with multiorgan involvement including, otherwise unexplained, severe rhabdomyolysis, metabolic acidosis, acute renal dysfunction, cardiac arrhythmias, hypotension, hyperkalemia, hepatomegaly, lipemia, fever, and death (1–6).

In the veterinary literature, there are limited reports documenting acute life-threatening perioperative hyperkalemia, for which the etiology is often unknown (7–14). The suspected causes have included low basal aldosterone levels (7), insulin resistance and mitochondrial dysfunction (8, 9), the use of alpha 2 agonist (7, 10–14) or propofol [drugs; (8, 15)]. A single case report by Mallard et al. (15) describes the clinical syndrome of rhabdomyolysis, myoglobinuria, metabolic acidosis, cardiac arrhythmias, increased level of liver enzyme, and methemoglobinemia in a dog receiving a continuous propofol infusion, suggestive of PRIS.

To the best of the authors’ knowledge, this is the first case report to suggest a PRIS-like syndrome in a domestic cat; with a development of perioperative severe hyperkalemia, ventricular fibrillation, rhabdomyolysis, and acute kidney injury.

## Case report

A six-month-old intact male domestic short-hair cat weighing 4.5 kg was admitted to our hospital as a referral for failing the outpatient treatment (subcutaneous fluids and maropitant^1^) and a two-day history of vomiting. Initial abdominal radiographs taken by the primary veterinarian did not show evidence of a gastrointestinal obstruction. Despite therapy, the patient continued to vomit.

On admission to our hospital, the cat’s vital signs were within normal limits, and the physical examination revealed tacky mucous membranes with slightly decreased skin turgor. Point-of-care blood work^2^ showed hyponatremia, hypochloremia, severe hyperlactatemia, mild azotemia, and severe hemoconcentration (Table 1). A complete blood count and full chemistry profile were not repeated, as the blood work performed 1 day prior by the referring veterinarian was normal, apart from mild elevations in amylase 1,167 U/L (Reference Interval(RI): 300–1,100 U/L) and Blood urea nitrogen (BUN) 46 mg/dl (RI: 10–30 mg/dl).

**Table 1:** Serial blood gas analysis, electrolytes, kidney, and glucose values.

	**Triage**	**1 h before inducing anesthesia**	**Intra op**	**Immediate postop**	**6 h postop**	**12 h postop**	**24 h postop**	**48 h postop**	**Reference interval**
PO_2_ (mmHg)	47.5	40.2	280.9	91.2	65.5	46.9	41.6	56.4	27.0–50.0
PCO_2_ (mmHg)	47.7	38.2	45.4	63.1	81.8	50.8	48.5	59.5	31.0–51.0
HCO_3_ (mmol/L)	29.3	31.5	25	18.7	27.6	27.8	27.0	27.4	15.0–27.0
pH	7.39	7.52	7.34	7.0	7.13	7.34	7.35	7.27	7.25–7.43
BE (mmol/L)	4.5	8.7	−0.7	−11.3	−1.5	2.1	1.5	0.5	−5.0 – 2.0
Sodium (mmol/L)	142	141	127	129	142	142	149	148	148–164
Potassium (mmol/L)	3.8	3.5	9	4.2	3.4	4.2	4	3.7	3.6–5.6
Chloride (mmol/L)	99	95	100	99	104	105	114	113	111–120
Ionized Calcium (mmol/L)	1.07	1.06	0.87	1.54	1.26	1.1	1.24	1.24	1.21–1.51
Lactate (mmol/L)	7.49	2.86	2.84	7.66	1.88	1.61	0.68	0.52	0.5–3.2
BUN (mg/dl)	52	49	>120	> 120	85	66	21	15	15–32
Creatinine (mg/dl)	1.53	1.49	7.58	7.63	2.94	2.27	0.95	0.79	0.5–1.9
Glucose (mg/dl)	141	133	375	573	338	167	185	164	63–133
Hematocrit (%)	67	52	56	48	54	49	36	35	28–50

A left peripheral cephalic catheter was inserted, and the patient was given a 5 ml/kg fluid bolus of Lactated Ringer’s solution (LRS)^3^ intravenously and continued on LRS with 20 mEq KCl/L^4^ at a rate of 90 ml/kg/day, not waiting for an abdominal ultrasound. Furthermore, the patient received maropitant (1 mg/kg intravenously), gabapentin^5^ (10 mg/kg orally), and buprenorphine^6^ (0.01 mg/kg intravenously) for anxiolysis and pain control.

A small bowel obstruction was confirmed in the abdominal ultrasound, and abdominal exploratory surgery with castration was recommended, given its intact reproductive status. The patient was maintained on LRS with 20 mEq KCl/L before surgery. A repeat venous blood gas analysis 1 h before inducing anesthesia showed persistent, mild hyponatremia and hypochloremia, new mild hypokalemia, resolved hyperlactatemia, persistent mild azotemia, and improved hemoconcentration (Table 1).

Since the cat had clinically improved, it was decided to proceed with the surgery. The patient had normal signs before inducing anesthesia; however, as a precautionary measure, treatment was considered. The cat was premedicated with midazolam^7^ (0.2 mg/kg, intravenously), ketamine^8^ (1 mg/kg, intravenously), and buprenorphine (0.02 mg/kg, intravenously). An electrocardiogram (ECG)^9^ before inducing anesthesia showed a normal sinus rhythm with a heart rate of 180 beats/min. Propofol^10^ (3 mg/kg, intravenously) was administered for anesthesia, following 5 min of preoxygenation. The cat was orotracheally intubated and maintained on sevoflurane^11^ (1.5–3 vol %). Subsequently, a nasogastric tube^12^ was inserted, and the stomach decompressed because the cat regurgitated during intubation. During anesthesia, monitoring was done for lingual pulse oximetry,^13^ ECG, capnography,^14^ rectal temperature, and continuous oscillometric blood pressure.^15^

Lactated Ringer’s Solution (5 ml/kg/h, intravenously) was administered throughout the perioperative period. Potassium supplementation was not incorporated into the fluids during anesthesia. During surgical preparation, no arrhythmias were noted and end-tidal carbon dioxide (CO2) was appropriate at 35 mmHg. Cefazolin^16^ (22 mg/kg, intravenously) was administered for prophylactic antimicrobial treatment during surgery. An ultrasound-guided^17^ local anesthetic blockade of the transversus abdominis plane (TAP) was performed. It comprised (in total) of bupivacaine^18^ (1 mg/kg), ketamine (1 mg/kg), and dexmedetomidine^19^ (1 mcg/kg), and was injected bilaterally along the intended ventral abdominal surgical site for regional anesthesia.

Upon moving to the operating room, the patient was noted to be mildly hypertensive with a mean arterial pressure (MAP) of 135 mmHg and tachycardic (180 bpm). An additional dose of ketamine (2 mg/kg, intravenously) was administered, and the patient’s heart rate and MAP improved to 150 bpm and 90 mmHg, respectively. After initial improvement, the patient became apneic and was transitioned onto the ventilator. Furthermore, new hypotension developed, and a 5 ml/kg LRS fluid bolus was given. The MAP normalized to 85 mmHg. Approximately 45 min after inducing anesthesia, the patient developed an apparent acute ventricular arrhythmia with a rate of 200 bpm. A lidocaine^20^ (0.25 mg/kg) bolus was administered intravenously and a temporary conversion to normal sinus rhythm occurred. Blood from the left lateral saphenous vein was collected for blood gas analysis during this time, and the patient’s temperature was noted to be 99𝔯.

A ventricular arrhythmia recurred with a rate of 200 bpm, after 1–2 min of normal sinus rhythm. The lidocaine (0.25 mg/kg) bolus was administered, followed by an additional 5 mL/kg LRS fluid bolus. Shortly after administering lidocaine, Torsades de Pointes was identified on ECG. Magnesium sulfate^21^ (0.15 mEq/kg) was administered intravenously as a bolus. However, the heart rhythm ultimately devolved into ventricular fibrillation.

Cardiopulmonary resuscitation was immediately initiated with external chest compressions while the diaphragm was opened to allow for internal chest compressions. Sevoflurane was discontinued. The rescue drugs including atropine^22^ (0.04 mg/kg), epinephrine^23^ (0.01 mg/kg), naloxone^24^ (0.04 mg/kg), and atipamezole^25^ (100 mcg/kg) were administrated intravenously. Internal defibrillation^26^^,^^27^ was performed at 2 joules (0.44 J/kg), the lowest joules allowable by the defibrillator, and the patient was successfully converted to a normal sinus rhythm.

A repeat of venous blood gas analysis showed worsening, moderate hyponatremia, new severe hyperkalemia, persistent hypochloremia, new severe azotemia, severe hyperglycemia, and persistent hemoconcentration (Table 1). The hyperkalemia was immediately treated with intravenous calcium gluconate^28^ (1 mL/kg) over 5 min, short-acting insulin^29^ (1 unit IV), dextrose 50%^30^ (1 mL/kg) diluted 1:1 with sterile saline, albuterol^31^ puff through the endotracheal tube, and terbutaline^32^ (0.01 mg/kg, IV). The treatment for hyperkalemia was repeated approximately 20 min later when bradycardia was detected and a decrease in blood pressure was noted.

After returning to a normal sinus rhythm, the abdominal exploration was continued and the foreign object was removed via gastrotomy. A chest tube^33^ was placed through the left body wall and the diaphragm was closed. After resuscitation, and for the remainder of surgery, the patient had mydriatic, non-responsive pupils and demonstrated periods of neurogenic breathing characterized by agonal, jerking, and ineffective breathing efforts, possibly related to anoxic brain injury.

Upon completion of the surgery, a triple lumen central line catheter^34^ was placed in the left jugular catheter. The patient was simultaneously castrated to limit the need for future anesthesia, and a urinary catheter^35^ was placed to monitor the urine production due to the new acute kidney injury.

The neurologic status of the patient slowly improved during the immediate postoperative period. His pupillary light response (PLR) and pupil size improved and he began breathing spontaneously. He maintained a normal sinus rhythm, though mild hypotension was noted. An additional fluid bolus (5 ml/kg) was administered, and his blood pressure was normalized.

Upon transfer to the intensive care unit, a repeat of blood gas analysis showed persistent moderate hyponatremia, resolved hyperkalemia, persistent azotemia, severe hyperglycemia, and normal hematocrit (Table 1). The in-house level for creatine kinase (CK) was 2,528 U/L (Table 2).

**Table 2:** Serial creatine kinase values.

	**Post op**	**1 day postop**	**2 days postop**	**3 days postop**	**5 days postop**	**Reference interval**
Creatine Kinase (U/L)	2,528					0–314 U/L *
Creatine Kinase (IU/L)		30,767	7,453	4,768	227	56–529 IU/L**

* In-house creatine catalyst. ** Creatine Kinase Catalyst, ANTECH Laboratories, North New Hyde Park, NY.

During the immediate postoperative period, the patient was maintained on LRS with 2.5% dextrose supplementation. Since there were concerns of a drug reaction leading to his anesthetic crisis, the patient was not administered any additional cefazolin, midazolam, dexmedetomidine, ketamine, buprenorphine, and propofol for the remainder of the day. The patient was started on a continuous rate infusion of fentanyl^36^ (3 mcg/kg/h) for postoperative analgesia. The patient was monitored closely with continuous telemetry, serial blood pressure, blood glucose, and the Modified Glasgow Coma Scale (MGCS). A single dose of hypertonic saline^37^ (2 mL/kg) was given intravenously due to suspected Cushing reflex, and ampicillin/sulbactam^38^ (30 mg/kg) was administered due to concerns that sterility was not maintained during the operation.

A repeat blood gas analysis performed approximately 6 h after surgery showed improved, mild hyponatremia, new mild hypokalemia, improving hypochloremia, improved but elevated BUN, normal creatinine, moderate hyperglycemia, and recurrent hyperlactatemia with relapsing hemoconcentration (Table 1). The patient received an additional 10 ml/kg LRS bolus. A repeat blood gas analysis performed 12 h after surgery showed normal potassium, improved azotemia, and resolved hyperlactatemia (Table 1). The patient’s vitals remained stable overnight, and his MGCS score was improving, though he became progressively delirious, agitated, and began rolling like an alligator. A dexmedetomidine constant rate infusion (CRI) at 1 mcg/kg/h and a single ketamine bolus (1 mg/kg, intravenously) was started due to the agitation and the need to preserve his instrumentation. There were no signs of adverse effects.

A repeat of blood gas analysis performed approximately 24 h after surgery showed resolved azotemia with hyperglycemia (Table 1). A repeat of the in-house CK level was attempted; however, because the value exceeded the high end of the reference interval the blood was sent to an outside laboratory^39^ and was reported as 30,767 IU/L (Table 2). Since the patient was moderately hyperglycemic, nonazotemic, and euhydrated, the dextrose supplementation was discontinued, and the fluid rate decreased to 50 ml/kg/day.

The physical examination revealed the patient to be cortically blind, and despite dexmedetomidine, he continued to be agitated, ataxic, and attempting to remove his catheters. A midazolam infusion was re-introduced at 0.1 mg/kg/h. In addition, gabapentin (21 mg/kg) and trazodone^40^ (7.5 mg/kg) were given enterally via the nasogastric tube for further sedation. In addition, a metoclopramide^41^ infusion at 2 mg/kg/day and Royal Canine Feline Renal^42^ liquid nutrition at one-fourth of the cat’s resting energy requirement were started through the nasogastric tube.

The patient’s electrolyte abnormalities and azotemia were resolved entirely during the second day, post-surgery (Table 1). A repeat CK was 7,453 IU/L (Table 2). Since there was a clinical improvement, the patient’s treatments and instrumentation were de-escalated. A gross myoglobinuria was not detected visually. Although a urinalysis was not performed, the cat’s urine production throughout hospitalization ranged from 1.1–3 ml/kg/h. The patient was weaned off all infusions except for fentanyl for pain control. The cat continued his recovery in a padded cage due to the ongoing, although improving, dysphoria, agitation, blindness, and ataxia. The patient ate well when food was offered in front of his face.

The cat was discharged on the 3rd day, post-surgery due to financial considerations. A repeat of CK in an outside laboratory, at discharge, was 4,768 IU/L (Table 2). The patient continued to be cortically blind; however, he could walk and stand continuously for several minutes. During a 2-day follow-up appointment, the owner reported the cat to be mildly mentally altered, ataxic with occasional circling, non-visual, and urinating outside the litter box; however, he was eating, drinking, and affectionate. A repeat of CK performed by an outside laboratory was 227 IU/L (Table 2).

Approximately 5 months post-hospitalization, while writing this report, the owner reports the cat is thriving. As per the owner, the cat plays, jumps, and interacts well within the household. The cat appears to have regained his vision, and there are no remaining neurologic deficits. The owner considers the cat neurologically and mentally back to normal.

## Discussion

To the best of the authors’ knowledge, this report is the first to document the development of rhabdomyolysis, severe hyperkalemia, cardiac arrhythmia, and acute kidney injury during general anesthesia in a domestic cat, suggestive of PRIS.

PRIS is a life-threatening complication secondary to propofol administration that has been reported in the human literature since the 1990s (5). PRIS has been typically associated with long-term (>48 h) and high-dose (>4 mg/kg/h) infusions of propofol (2, 16). However, numerous reports in human medicine (16–19) and a case report of recurrent, suspected propofol-associated hyperkalemia in a dog (8) have described PRIS syndrome after single bolus dosing or at lower doses, or shorter time courses than previously thought. A universally accepted definition for PRIS has not been established, but the pathophysiology is likely multifactorial and complex. At the intracellular level, propofol impairs oxygen utilization and inhibits electron flow along the mitochondrial electron transport chain in skeletal and cardiac muscle cells, ultimately leading to decreased ATP production, cellular hypoxia, and cell death (20). The clinical spectrum is characterized by sudden and unexplained rhabdomyolysis, metabolic acidosis, cardiac arrhythmias, hyperkalemia, AKI, multiorgan failure, and death (1–6). The mortality rates have been reported as high as 74% (6).

Veterinary medicine presents limited cases documenting the development of perioperative hyperkalemia after administration of propofol (8, 15) with one confirmed (15) case consistent with PRIS in a dog. Our patient shares many clinical and biochemical characteristics that are associated with PRIS in humans, and similar to that of the Mallard case report (15). Our patient had a very mild azotemia, suspected to be pre-renal, and mild hypokalemia, before the administration of a single bolus of propofol. The ensuing cardiac arrhythmia, hyperkalemia, severe elevation in CK, and marked azotemia developed acutely after administering propofol and was resolved after aggressive treatment, without recurrence, even after reintroduction of all other pre-operative medications other than propofol and cefazolin.

Various hypotheses for the development of perioperative hyperkalemia during general anesthesia have been proposed. They include possible drug-related causes including the administration of medications that alter the clearance of potassium or that can alter its homeostasis, familial or breed-associated endocrine or metabolic causes such as low aldosterone levels, mitochondrial dysfunction, renal impairment, or trauma from surgical procedure (7–15, 21). While all these were considered in our case, several could be ruled out early. Our patient was not chronically taking medications that would alter potassium excretion (i.e., ACE inhibitors or potassium-sparing diuretics), and even though mild azotemia was present before surgery, it was not severe enough to question whether renal insufficiency may limit potassium excretion. Furthermore, the patient’s urine production postoperatively was within normal limits relative to his fluid administration. Our patient also had a normal body temperature at the time of crisis during anesthesia, ruling out malignant hyperthermia as a cause of his rhabdomyolysis. Hypoaldosteronism is rare in cats (22) and while basal cortisol or aldosterone levels were not tested, it was not suspected in our patient as he did not have a clinical history suggestive of systemic illness, and the patient’s sodium-to-potassium ratio at the time of admission was greater than 37.

Because of the administration of multiple perioperative local analgesic and anesthetic agents, there was a possibility of perioperative medication-induced hyperkalemia. However, several studies of interest have suggested dexmedetomidine may play a contributing role (7, 10–14). Hyperkalemia caused by alpha 2 agonists is hypothesized to be induced by the inhibitory effects of the alpha 2-adrenergic receptor on insulin production which can alter the potassium homeostasis (14). These inhibitory effects were hypothesized to be the contributing factors in anesthesia-induced hyperkalemia in a study of 11 captive non-domesticated felids (23) and in a retrospective study (10) evaluating the development of acute hyperkalemia in 19 client-owned dogs. However, hyperkalemia due to a combined effect of gas inhalant and alpha 2 agonists was also a possibility in that study (10).

In our case, the patient had confirmed mild hypokalemia just before being induced general anesthesia. He did not receive any drugs in the peri-anesthetic period that would impair potassium excretion, and the intraoperative fluids were verified to have no potassium-containing additives. An alpha 2 agonist (dexmedetomidine) was administered at a low dose as part of local anesthesia, but there was no suspicion of accidental systemic administration as no blood was aspirated from the injection needle during the local block. While local dexmedetomidine is absorbed systemically over time and delayed adverse effects are possible, we do not suspect this medication played a role in the intra-operative hyperkalemia encountered in our patient. Due to profound dysphoria and agitation in the postoperative period, our patient was placed on a continuous infusion of dexmedetomidine at the same initial dose given locally but now delivered intravenously for 28 h, without recurrent complications or recurrent rises in potassium.

In addition to the marked hyperkalemia, our patient had documented marked elevations in CK values with a concurrent acute kidney injury, consistent with rhabdomyolysis, despite no reported gross myoglobinuria. An elevated plasma CK level is the most sensitive laboratory finding pertaining to rhabdomyolysis and it is the standard biomarker for diagnosis (24, 25). The subsequent hyperkalemia and acute renal failure that occur with rhabdomyolysis represent the major life-threatening complications (24). Myoglobin is excreted in the urine following muscle damage, when the myoglobin levels exceed the plasma protein binding capacity, the renal threshold, and can lead to the production of tea-colored urine (25). In humans, myoglobin has a half-life of 2–3 h, which is much shorter than the half-life of CK, which is 36 h. Because of the short half-life of myoglobin, it is not uncommon for myoglobinuria to go unnoticed. Its presence may be affected by many factors or may be resolved before urinary testing, and its absence does not exclude diagnosis (24, 25). Rhabdomyolysis is diagnosed when CK exceeds 5 times the upper limit of normal (25), which was detected in our patient. A urinary catheter was placed in our patient to monitor urinary outputs postoperatively due to the new azotemia and acute kidney injury. However, we had missed performing a urinalysis to provide further evidence and support of rhabdomyolysis when it was suspected.

The surgery and CPR with defibrillation may also have contributed to the development of elevated CK levels in our patient. However, the magnitude of this elevation is unlikely to have been due to these events alone. Human studies have evaluated the effects of various surgeries, including intrathoracic and intraabdominal surgeries, and CPR on CK levels and have found the maximum CK level to be 1,339 IU/L (RI 10–180 IU/L) 2 days postoperative after extensive muscle surgery (26) and 2,984 + 701 IU/L (49.1 + 11.7 IU/L) after CPR and external defibrillation (27). While no veterinary studies have been published, these values are far below the 30,767 IU/L seen in our patients. In the case report by Mallard et al., a maximum CK level of 37,941 IU/L was documented 2 days after propofol administration (15). While the cause of elevated CK levels in our patient is likely multifactorial, the maximum elevation in CK was documented within 24 h of surgery, suggesting a more immediate cause of the marked elevation and further supporting the potential of propofol-induced rhabdomyolysis.

It has been hypothesized that hereditary mitochondrial fatty acid metabolism impairment may be responsible for susceptibility to PRIS in some human patients (28, 29). It is not known if our patient had hereditary errors of fatty acid oxidation as this was not investigated. Low carbohydrate supply has been implicated as a risk factor for PRIS, due to increased lipolysis during times of starvation (29). Stress and endogenous catecholamine and glucocorticoid release are also responsible for the regulation of lipolysis (1). The free fatty acids released from lipolysis undergo beta-oxidation in the mitochondria and become the principal energy source in skeletal muscle and cardiovascular cells. Propofol can hinder this pathway, further inhibiting ATP production and contributing to cell death and necrosis (1). Adequate carbohydrate intake is recommended to suppress lipolysis in periods of starvation, and a glucose infusion is reported to be sufficient to reduce endogenous lipolysis (29).

Our patient was inappetant for at least 3 days before surgery and it is likely his carbohydrate reserves were low, leading to the onset of lipolysis for energy. Furthermore, while serum cortisol and catecholamine levels were not measured, a degree of behavioral and physiologic stress is expected considering the obstruction by the foreign object. Our patient was administered dextrose as a bolus as well as supplementation in his fluids to treat his hyperkalemia which may have helped contribute to his resolution of suspected PRIS.

While there are several differences between our case compared to cases of suspected PRIS reported in human and veterinary literature, the timing of events and the patient’s clinical course is suggestive of an adverse effect of propofol. However, it is not known if this patient had other extenuating metabolic circumstances making him more susceptible to the development of severe, acute hyperkalemia. In addition, since other medications were given, these may have also played a role, thereby limiting our ability to implicate propofol with certainty. Furthermore, the majority of reported cases of PRIS occur after long-term, high-dose propofol infusion, whereas our patient received propofol as a single, bolus dose, though reports of suspected PRIS of this nature exist (8, 16–19). It would have been advantageous to have completed a urinalysis to further support the diagnosis of rhabdomyolysis, which is one of the characteristics of PRIS and the highest suspected cause of hyperkalemia in this case.

To the best of the authors’ knowledge, this is the first case report suggesting PRIS in a domestic cat. While a definitive diagnosis was not possible, the association between the administration of propofol and the cat’s development of rhabdomyolysis, hyperkalemia, cardiac arrhythmia, and acute kidney injury without any other apparent cause makes a causal relationship possible. As and when additional information on PRIS is generated, the identification of these events may become more likely. Veterinarians should be aware of this potential complication of propofol administration, which may not only occur in young and critically ill humans but can potentially occur in our patient populations as well.

## Data availability statement

The original contributions presented in the study are included in the article/supplementary material, further inquiries can be directed to the corresponding author.

## Ethics statement

Written informed consent was obtained from the owners for the publication of this study.

## Author contributions

TI: Writing – original draft. SG: Writing – review & editing.

## Footnotes

^1^Maropitant Citrate Injection Solution, 10 mg/ml, Zoetic Inc., Kalamazoo, MI.

^2^Element POC Blood Gas & Electrolyte Analyzer, HESKA, Loveland, Colorado 80,538.

^3^Lactated Ringers 1 L Bag, Baxter Healthcare Corporation Deerfield IL 60015.

^4^Potassium Chloride Injection, 40 mEq/20 ml, Fresenius Kabi, Lake Zurich, IL.

^5^Gabapentin Suspension, 50 mg/ml, Wedgewood Pharmacy LLC, Swedesboro, NJ

^6^Buprenorphine Hydrochloride, 0.3 mg/ml, Hospira, Inc., Lake Forest, IL.

^7^Midazolam Injection, 50 mg/10 ml, Almaject Inc., Morristown, NJ.

^8^Ketamine Hydrochloride Injection, 100 mg/ml, Vet One, Boise, ID.

^9^Lead II ECG, SurgiVet, Smiths Medical ASD, INC St. Paul, MN.

^10^Propofol Injection, 10 mg/ml, SAGENT Pharmaceuticals, Schaumburg, IL.

^11^Petrem Sevoflurane USP, 250 ml, Piramal Critical Care Inc., Bethlehem, PA.

^12^MILA Nasogastric Feeding Tube Plus with Stylet, 5 Fr x 22″, MILA International Medical Instrumentation for Animals Florence, Kentucky.

^13^SpO2 Model 9,847 V, NONIN PureSAT, Plymouth, Minnesota.

^14^Capnography, SurgiVet, Smiths Medical ASD, INC St. Paul, MN.

^15^Cardell Insight Veterinary Diagnostic Monitor, Midmark, Versailles, OH.

^16^Cefazolin Injection, 100 mg/ml, Hikma Pharmaceuticals, Eatontown, NJ.

^17^SonoQue V1.0.0 (Ipad Air Version 15.31), Yorba Linda, CA.

^18^Bupivacaine Injection, 5 mg/ml, Hikma Pharmaceuticals, Berkeley Heights, NJ.

^19^Dexmedetomidine hydrochloride, 0.5 mg/ml, Dechra Veterinary Products, Overland Parks, KS.

^20^Lidocaine 2% Injection, Covetrus, MWI, Boise, ID

^21^Magnesium Sulfate Injection USP, 5 g/10 ml, Fresenius Kabi, Lake Zurich, IL.

^22^Atropine Sulfate Injection, 0.54 mg/ml, MWI, Boise, ID.

^23^Epinephrine Injection 1 mg/ml VEDCO, INC St. Joseph MO.

^24^Naloxone HCI Injection USP, 4 mg/20 ml, Akorn Inc., Lake Forest, IL.

^25^Revertidine Injection, 5.0 mg/ml, Modern Veterinary Therapeutics, Miami, Florida.

^26^Sterilizable Internal Handle w/ Integrated Paddles, Without Switch, Zoro Tools, Buffalo Grove.

^27^HEWLETT PACKARD REF M2475B Codemaster XL No battery * paddle.

^28^Calcium Gluconate Injection USP, 1000 mg/10 mL, Fresenius Kabi, Lake Zurich, IL.

^29^Novolin R Insulin, Novo Nordisk Inc., Plainsboro, NJ.

^30^Dextrose Solution 50%, Phoenix Pharmeceutical Inc., St. Joseph, MO.

^31^Albuterol Sulfate HFA Inhalation Aerosol 90 mcg per actuation, Teva Pharmaceutical Industries Ltd., El Dorado Hills, CA.

^32^Terbutaline Sulfate Injection USP, 1 mg/mL, Hikma Pharmaceuticals, Eatontown, NJ.

^33^Chest Tube Guidewire Inserted, 12 gauge x 30 cm, MILA International Medical Instrumentation for Animals Florence, Kentucky.

^34^Small Animal Long Term Venous Catheterization Set Seldinger Technique Guidewire Introducer, 5 FrT 13 cm Florence, Kentucky.

^35^Kendall Feeding Tube and Urethral Catheter, 3.5 FrT, Covidien IlC, Mansfield, MA.

^36^Fentanyl Citrate Injection, 2,500 mcg/50 ml, Hikma Pharmaceuticals, Berkeley Heights, NJ.

^37^Hypertonic Saline 7.2%, VEDCO INC, St. Joseph, MO.

^38^Ampicillin and Sulbactam Injection USP, 3 g/vial, Auromedics, E. Windsor, NJ.

^39^Creatine Kinase Catalyst, ANTECH Laboratories, North New Hyde Park, NY.

^40^Trazodone Hydrochloride Tablet USP, 50 mg, Torrent Pharmaceuticals LTP, Levittown, PA.

^41^Metoclopramide 10 mg/2 mL, Hospira, INC, Lake Forest IL.

^42^Royal Canine Veterinary Diet Feline Renal Support Liquid, DHKS Pet Supplies Pte Ltd.
